# Initial single-institutional experience with salvage surgery for stage IV non-small-cell lung cancer

**DOI:** 10.1093/icvts/ivaf029

**Published:** 2025-02-14

**Authors:** Tomoyuki Hishida, Naoyuki Oka, Kaito Yano, Seiji Omura, Yu Okubo, Kyohei Masai, Kaoru Kaseda, Keiko Ohgino, Hideki Terai, Hiroyuki Yasuda, Keisuke Asakura

**Affiliations:** Division of Thoracic Surgery, Department of Surgery, Keio University School of Medicine, Tokyo, Japan; Division of Thoracic Surgery, Department of Surgery, Keio University School of Medicine, Tokyo, Japan; Division of Thoracic Surgery, Department of Surgery, Keio University School of Medicine, Tokyo, Japan; Division of Thoracic Surgery, Department of Surgery, Keio University School of Medicine, Tokyo, Japan; Division of Thoracic Surgery, Department of Surgery, Keio University School of Medicine, Tokyo, Japan; Division of Thoracic Surgery, Department of Surgery, Keio University School of Medicine, Tokyo, Japan; Division of Thoracic Surgery, Department of Surgery, Keio University School of Medicine, Tokyo, Japan; Division of Respiratory Medicine, Department of Internal Medicine, Keio University School of Medicine, Tokyo, Japan; Division of Respiratory Medicine, Department of Internal Medicine, Keio University School of Medicine, Tokyo, Japan; Division of Respiratory Medicine, Department of Internal Medicine, Keio University School of Medicine, Tokyo, Japan; Division of Thoracic Surgery, Department of Surgery, Keio University School of Medicine, Tokyo, Japan

**Keywords:** non-small cell lung cancer, salvage surgery, systemic therapy, stage IV, tyrosine kinase inhibitor, cytotoxic agents, immune checkpoint inhibitors

## Abstract

The purpose of this study was to assess surgical outcomes of salvage surgery for clinical stage IV non-small-cell lung cancer. A total of 14 patients who underwent lung resection following systemic therapy between 2010 and 2022 were included in this study. Systemic therapy prior to surgery included agents including epidermal growth factor receptor tyrosine kinase inhibitors (EGFR-TKIs) in eight patients and non-TKI agents in six (chemotherapy alone: four, chemotherapy plus immune checkpoint inhibitors: two). During a median follow-up of 5.2 years, the EGFR-TKI group showed a favourable 5-year overall survival of 83%; however, it was due to treatment after relapse, and there were no 4-year relapse-free survivors. The non-EGFR-TKI group showed a 5-year relapse-free survival of 33%, and 2 patients have survived more than 3 years without any relapse and further treatment. When considering the role of surgery in multimodal treatment for initial c-stage IV non-small-cell lung cancer, salvage surgery following non-TKI therapy (chemotherapy with or without immune checkpoint inhibitor) can be regarded as genuine salvage surgery.

## INTRODUCTION

Salvage surgery for non-small-cell lung cancer (NSCLC) has traditionally been indicated for patients with loco-regional residual or recurrent lesions due to failure after definitive chemoradiotherapy for loco-regional (clinical [c-] stage III) disease [1, 2]. Recently, due to the advances in systemic therapy, including targeted therapy and immunotherapy, selected patients with c-stage IV NSCLC experience similar outcomes as those with c-stage III disease after systemic therapy [3–5]. Salvage surgery may be considered as an effective treatment strategy even for c-stage IV disease; however, few studies have evaluated the surgical outcomes for this population. The objective of this study was to review our initial experience and to discuss the role of salvage surgery in multimodal treatment for initial c-stage IV NSCLC.

## PATIENTS AND METHODS

We reviewed clinicopathological records of 2096 patients who underwent pulmonary resection for primary lung cancer in our institution between 2010 and 2022. Among them, 14 patients (0.7%) underwent salvage surgery following systemic therapy for initial c-stage IVA-B NSCLC and were enrolled in this study. Salvage surgery was indicated for patients with locally residual or regrowing primary lesions after systemic therapy. The basic inclusion criteria were ycM0 status after systemic therapy and adequate physical fitness. Before planning surgery, all patients underwent whole-body examinations, including integrated positron emission tomography and computed tomography. This study was approved by the institutional ethics committee in 2023 (approval no. 20231021), and informed consent from each patient was waived due to the retrospective nature of the study.

## RESULTS

Patient characteristics and surgical outcomes are summarized in Table 1. The predominant histology at final pathology was adenocarcinoma in 12 patients (86%), and 9 had sensitizing epidermal growth factor receptor (*EGFR*) mutations (exon19 del or L858R). The most common M factor was M1b (*n* = 8, 71%), indicating an isolated distant metastasis mainly in the bone (*n* = 6). Four M1a patients had pleural dissemination, which was apparent on CT scans. Among two M1c patients, one had a single metastasis each in the brain and bone, and the other had five in the bone. Prior to the initiation of first-line systemic therapy, one patient received stereotactic radiotherapy for a solitary brain metastasis, while another underwent resection and prosthesis replacement for an isolated femoral bone metastasis for symptomatic control. Before surgery, eight patients (58%) received *EGFR* tyrosine kinase inhibitor (EGFR-TKI) with or without cytotoxic chemotherapy (EGFR-TKI group), while six received cytotoxic chemotherapy with or without immune checkpoint inhibitors (ICIs) (pembrolizumab or atezolizumab, non-EGFR-TKI group). Regarding cytotoxic chemotherapy, the primary regimen was carboplatin-based and was administered to seven patients (pemetrexed: three, docetaxel: two, nab-paclitaxel: one, etoposide: one). Additionally, three patients received a cisplatin-based regimen (cisplatin + pemetrexed + bevacizumab). The pre- and postoperative clinical courses of each patient are detailed according to the type of systemic therapy in Supplementary Fig. S1.

**Table 1:** Patient characteristics and surgical outcomes

Characteristics	No. (%)
Median age at surgery (range), years	54.5 (44–77)
Gender	
Male	8 (57)
Smoking history	
Never	7 (50)
Histology^a^	
Adenocarcinoma	12 (86)
Squamous cell carcinoma	1 (8)
Large cell neuroendocrine carcinoma	1 (8)
Initial c-stage	
IVA	12 (86)
IVB	2 (14)
Initial cN-factor	
N0/1/2/3	2/5/4/3
Initial cM-factor	
M1a/b/c	4/8/2
Systemic therapy before surgery	
EGFR-TKI (alone)	4 (29)
EGFR-TKI and cytotoxic agent	4 (29)
Cytotoxic agent (alone)	4 (29)
Cytotoxic agent and immune checkpoint inhibitor	2 (13)
Median time from 1^st^ line treatment to surgery (range), months	16 (3–65)
Indication of salvage surgery	
Residual primary tumour	8 (57)
Progressed primary tumour	6 (43)
Mode of resection	
Lobectomy	12 (84)
Bi-lobectomy	1 (8)
Wedge resection	1 (8)
R0 resection	10 (71)
Median blood loss (range), ml	75 (10–505)
Median postoperative stay (range), days	7 (6–17)
Postoperative complication	1 (8)
90-day mortality	0
Postoperative systemic therapy	10 (71)
EGFR-TKI	7
Cytotoxic agents	3

a At final pathological diagnosis after salvage surgery.

**Figure 1:**
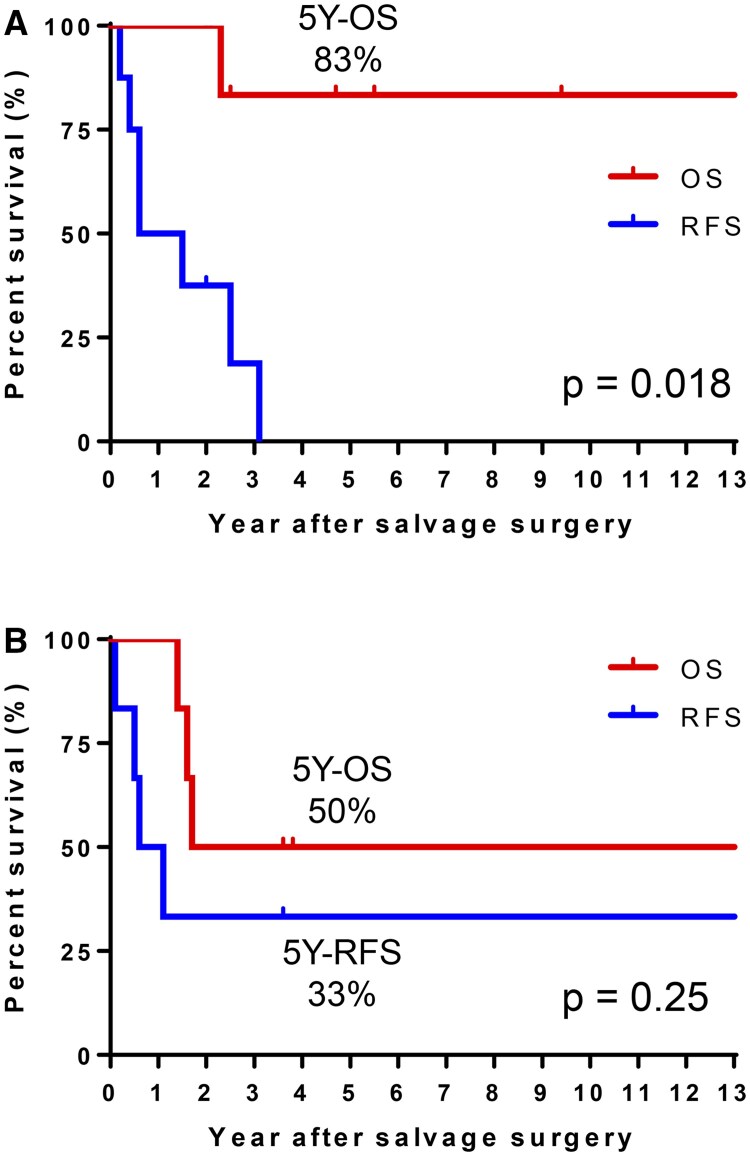
(**A**) Overall and recurrence-free survival (OS and RFS) after salvage surgery for patients who received EGFR-TKI preoperatively (EGFR-TKI group). The 5-year OS was favourable (83%); however, RFS was significantly worse than OS (*P* = 0.018, log-rank test). There were no 4-year RFS. (**B**) OS and RFS after salvage surgery for the non-EGFR-TKI group. The 5-year OS and RFS are 50% and 33%, respectively, which is not statistically different (*P* = 0.25).

In the EGFR-TKI group, six patients received first-generation EGFR-TKIs, such as gefitinib and erlotinib, and two received osimertinib just before surgery. The indication for salvage surgery was residual primary tumour in eight patients and progressed primary tumour in six patients. All but one patient demonstrated complete disappearance of all distant metastases (ycM0). One patient (patient 4) had a residual solitary adrenal metastasis, which was resected following salvage lung resection. For patients with pleural dissemination, its disappearance was confirmed using thin-section CT scans taken before surgery. All surgeries were performed using thoracotomy or video-assisted approach with mini-thoracotomy (≤8 cm). None of the cases were conducted using a robotic-assisted approach in this series. Lung resections were primarily performed by lobectomies (*n* = 12), with bronchial or vascular plasty conducted in three patients. Hilar/mediastinal nodal dissection was performed for patients undergoing lobectomy if there were no obstacles, including inflammatory changes around the lymph nodes. The median duration of surgery was 163 min (range, 111–291), and the median blood loss was 75 ml (range, 0–505). A pathological complete response was observed in one (patient 11) out of eight who underwent surgery for residual primary tumour. Four cases of R1/2 disease were found as residual pleural dissemination or positive pleural cytology. One patient (patient 11) developed grade 2 pneumonia postoperatively, but no 90-day mortality was observed. Postoperatively, seven of eight patients in the EGFR-TKI group continued with the same EGFR-TKI as before surgery. One patient (patient 3) initially received a cisplatin-based regimen after surgery but developed a relapse in the thoracic cavity within 2 months and subsequently received gefitinib. In the non-EGFR-TKI group, two of six patients received a cisplatin-based regimen postoperatively. Overall, 11 patients (79%) experienced relapse in distant lesions (*n* = 9) and ipsilateral hilar/mediastinal nodes (*n* = 2). The median follow-up period was 5.2 years, and the 5-year overall and relapse-free survival (OS and RFS) for the entire population was 70% and 18%, respectively. The EGFR-TKI group showed a 5-year OS of 83%. However, seven of the eight patients relapsed, resulting in a significantly worse RFS compared to OS (*P* = 0.018, log-rank test). There was no 4-year RFS (Fig. 1A), but five of the seven relapsed patients received osimertinib during treatment, leading to a favourable median post-relapse survival (PRS) of 38.9 months (range, 1.0–128.9). In contrast, the non-EGFR-TKI group had a 5-year OS of 50% and RFS of 33%, which was not statistically different (*P* = 0.25, Fig. 1B). Two patients (patients 9 and 11) have survived for more than 3 years without any relapse and additional treatment.

## DISCUSSION

This study highlighted the surgical outcomes of salvage surgery in patients with initial c-stage IV NSCLC. While we previously reported surgical outcomes of salvage surgery with various clinical stages [5], the present study focused specifically on a c-stage IV population in our institution. Notably, complex surgical procedures, such as bronchial and vascular plasty, were required in three patients; however, only one grade 2 pneumonia was observed, and there was no 90-day mortality. These favourable safety profiles can be attributed to the absence of prior thoracic irradiation.

Regarding postoperative survival, it is noteworthy that survival outcomes varied significantly based on the systemic therapy administered prior to surgery. The EGFR-TKI group showed a favourable 5-year OS of 83%, which can be attributed to a relatively longer PRS (median, 38.9 months) compared to reported PRS in the AURA3 trial after systemic therapy with platinum-pemetrexed or osimertinib for first or second generation EGFR-TKI-resistant NSCLC (median, 22.5–26.8 months) [6]. The observed discrepancy between unsatisfactory RFS and favourable PRS might be due to the generally cytostatic mechanism of TKIs. Ohtaki *et al*. examined 36 patients from multiple institutions with initial c-stage of III–IV who underwent salvage surgery following EGFR- or ALK-TKI treatment. They reported a favourable 3-year OS of 75.1%, but a low 3-year RFS of 22.2% [7]. Thus, salvage surgery alone has a limited effect in salvaging patients treated with EGFR-TKIs. For these patients, salvage surgery may be more appropriately considered as an adjunct or adjuvant to systemic therapy. In contrast, the non-EGFR-TKI group had an estimated 5-year RFS was 33%, with two of six patients surviving longer than 3 years without relapse and postoperative treatment. Selected patients treated with cytotoxic agents or ICIs may benefit from salvage surgery alone. The number of candidates for salvage surgery following ICI treatment is expected to increase. Further studies with larger sample sizes are warranted to validate these findings.

The current study was exploratory in nature, with limitations including the small sample size and the variety of systemic treatments used. Although the primary inclusion criteria were ycM0 status and good performance status, potential unknown selection biases may have been present. Additionally, 86% of the study cohort had initially c-IVA disease, which may have a lower tumour burden compared to IVB disease. Therefore, relatively favourable OS observed could also be influenced by the biological nature of the disease. To address these limitations, a multi-institutional prospective randomized study with a non-surgical control group and clearly defined inclusion criteria should ideally be considered to validate the current findings and clarify the survival benefit of surgery in the near future.

In conclusion, salvage lung resections following systemic therapy are feasible for patients with initial c-stage IV NSCLC. Survival profiles varied significantly depending on the type of systemic therapy prior to surgery (EGFR-TKI or chemotherapy/ICI). The EGFR-TKI group showed favourable OS but short RFS, while selected patients in the non-EGFR-TKI group achieved long-term RFS without postoperative therapy. When considering the role of surgery in multimodal treatment for patients with initial c-stage IV NSCLC, salvage surgery following non-TKI therapy (chemotherapy with or without ICI) can be regarded as genuine salvage surgery.

## Supplementary Material

ivaf029_Supplementary_Data

## Data Availability

The data underlying this article cannot be shared publicly due to the privacy of individuals that participated in the study. The data will be shared on reasonable request to the corresponding author.

## Notes

Presented as a virtual poster at the 2023 World Conference on Lung Cancer (WCLC), Singapore, 9–12 September 2023.
