# Supplementary material for: Initial single-institutional experience with salvage surgery for stage IV non-small-cell lung cancer
Source: Interdiscip Cardiovasc Thorac Surg. 2025 Feb 14;40(3):ivaf029. doi: 10.1093/icvts/ivaf029 (PMC11906397; doi:10.1093/icvts/ivaf029)
Supplement: ivaf029_Supplementary_Data [file ivaf029_supplementary_data.zip › 202407 salvage supplemental figure legend.docx]

**Supplemental Figure legend**

**Supplemental Fig. 1.** Swimmer plot of clinical courses of patients.

Pt, patient; His, histology; Ad, adenocarcinoma; Sq, squamous cell carcinoma; Lc, large cell neuroendocrine carcinoma; M, M-factor; diss, dissemination; adre, adrenal gland; br/bo, brain and bone; cerv, cervical lymph node; Ind, indication of salvage surgery; RE, residual primary tumor; PD, progressed disease of primary tumor; R, resection status; R0, macroscopic and microscopic complete resection; R1, microscopic incomplete resection; R2, macroscopic incomplete resection; EGFR-TKI, epidermal growth factor receptor tyrosine kinase inhibitor; ICI, immune checkpoint inhibitor; GEF, gefitinib; OSM, osimertinib; ERL, erlotinib; AFA, afatinib; PEMB, pembrolizumab; NIVO, nivolumab; ATZ, atezolizumab; RFS, recurrence-free survival; PRS, post-recurrence survival.
