# Supplementary figures and images for: Initial single-institutional experience with salvage surgery for stage IV non-small-cell lung cancer
Source: Interdiscip Cardiovasc Thorac Surg. 2025 Feb 14;40(3):ivaf029. doi: 10.1093/icvts/ivaf029 (PMC11906397; doi:10.1093/icvts/ivaf029)

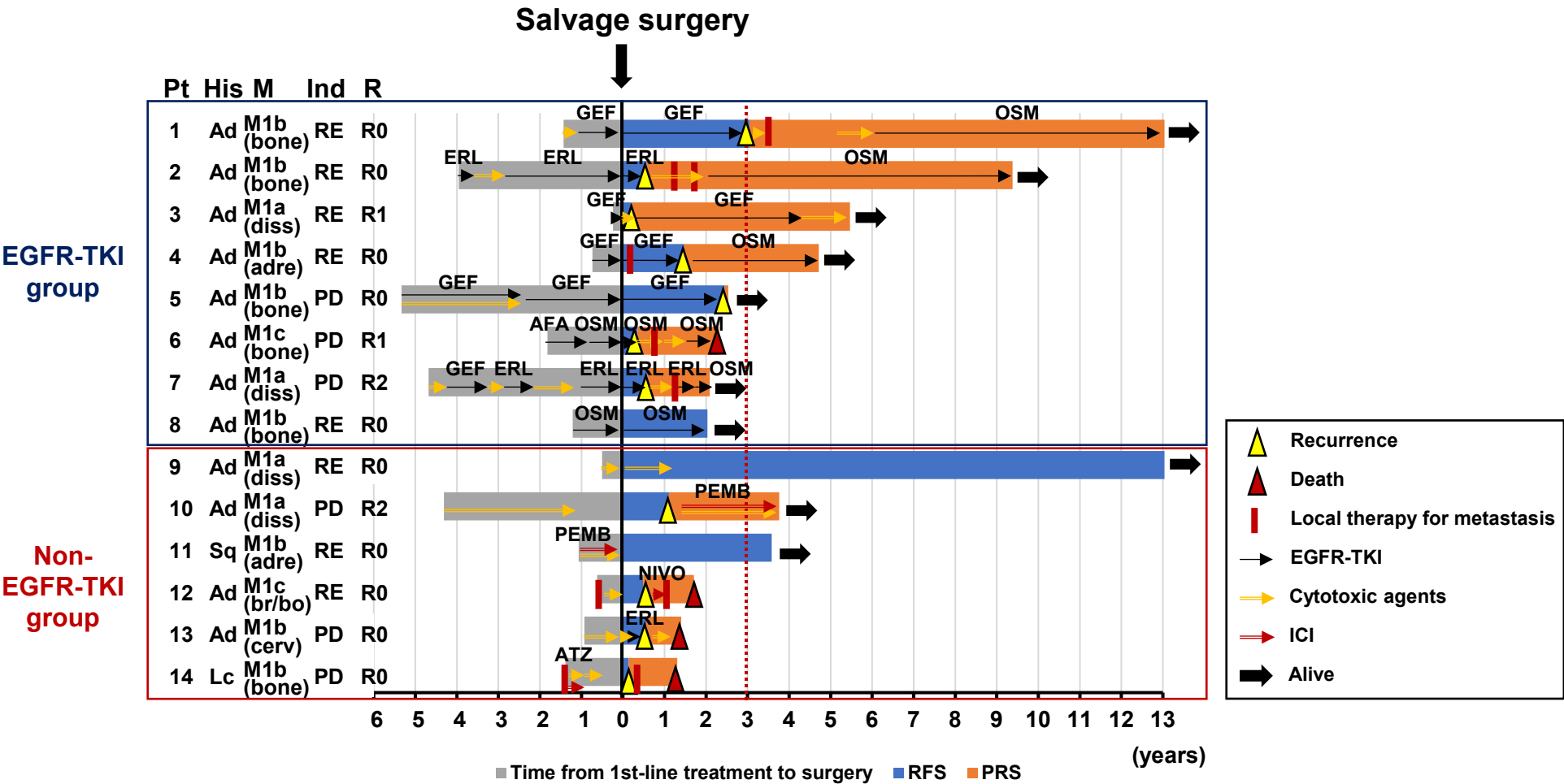

Supplemental Fig. 1

Supplement: ivaf029_Supplementary_Data [file ivaf029_supplementary_data.zip › Revised supplental figure 1R.pdf]
